# Cancer proliferation and therapy: the Warburg effect and quantum metabolism

**DOI:** 10.1186/1742-4682-7-2

**Published:** 2010-01-19

**Authors:** Lloyd A Demetrius, Johannes F Coy, Jack A Tuszynski

**Affiliations:** 1 Department of Organismic and Evolutionary Biology, Harvard University, Cambridge, MA 02138, USA; 2 TAVARLIN AG/TAVARGENIX GmbH, Landwehrstraße 54, 64293 Darmstadt, Germany; 3 Department of Experimental Oncology, Cross Cancer Institute, Edmonton, Alberta, Canada

## Abstract

**Background:**

Most cancer cells, in contrast to normal differentiated cells, rely on aerobic glycolysis instead of oxidative phosphorylation to generate metabolic energy, a phenomenon called the Warburg effect.

**Model:**

Quantum metabolism is an analytic theory of metabolic regulation which exploits the methodology of quantum mechanics to derive allometric rules relating cellular metabolic rate and cell size. This theory explains differences in the metabolic rates of cells utilizing OxPhos and cells utilizing glycolysis. This article appeals to an analytic relation between metabolic rate and evolutionary entropy - a demographic measure of Darwinian fitness - to: (a) provide an evolutionary rationale for the Warburg effect, and (b) propose methods based on entropic principles of natural selection for regulating the incidence of OxPhos and glycolysis in cancer cells.

**Conclusion:**

The regulatory interventions proposed on the basis of quantum metabolism have applications in therapeutic strategies to combat cancer. These procedures, based on metabolic regulation, are non-invasive, and complement the standard therapeutic methods involving radiation and chemotherapy

## Background

Cancer is an age-dependent disease characterized by five key hallmarks in cell physiology that drive the progressive change of normal differentiated cells into diverse states of malignancy [1]: autonomous growth-replication in the absence of growth signals; insensitivity to anti-growth signals; apoptosis-evasion of programmed cell death, angiogenesis-the induction of the growth of new blood vessels; invasion and metastasis

The age-dependency of cancer [2] and the relatively rare incidence of the disease during an average human life time suggest that adaptive mechanisms exist in cells and tissues to prevent this multi-step transition from a normal differentiated cell into malignancy. Consequently, each of these physiological changes constitutes the rupture of anti-tumor defences developed during the evolutionary history of the organism. It may be worth observing that a graph of the logarithm of the total cancer incidence against age approximates to a straight line with a gradient of 6-7 (the value of the power-law exponent) suggesting that 6-7 separate events are required for neoplastic transformation of a 'typical' human cell [3].

Cancer cells may be considered as autonomous units which have an impaired capacity to maintain the metabolic stability of the organism in which they reside. Anti-cancer therapies are corrective measures designed to remedy this impairment by eliminating the errant cells.

The nature of these corrective measures has undergone a significant development, starting with surgical resection of solid tumors followed by radiation and then chemotherapy, as the understanding of the biology of cancer has increased. The non-surgical therapies which came to dominate the treatment of the disease were based on the proposition that the disease is primarily the result of dynamic changes in the genome. This gene oriented perspective led to the notion that decoding the genetic instruction that determines the cancer phenotype would elucidate its origin and thus provide an effective biological basis for therapy. Genes represent the blueprint for phenotypic expression. Accordingly, the genetic model entailed therapies based on the complete elimination of cancer cells. Radiation and chemotherapy were the first class of anti-cancer strategies which this model invoked. These two therapeutic methods were designed to eliminate cancer cells from tissues. However, due to the low selectivity of this approach, non-cancer cells are also killed or damaged leading to severe side effects.

The next generation of cancer drugs which developed from this genomic viewpoint explicitly recognized the multi-step progression towards malignancy, and that in most instances death only occurs when the metastatic state is attained. Metastasis is often triggered by angiogenesis, the proliferation of a network of blood vessels that penetrates into cancerous tissue, supplying nutrients and oxygen [4].

Drugs that impede the formation of tumor blood vessels were therefore proposed as therapeutic agents in the combat against malignancy [5]. There exist, however, some disadvantages to this mode of therapy as angiogenic inhibitors sometimes trigger side effects and induce a more invasive type of tumor [6].

Studies in recent years have led to a re-evaluation of the genomic model of cancer and the development of a model based on cell metabolism [7]. The research which triggered this shift from genes to metabolic reactions was done in 1924 by Warburg [8] who recognized certain critical differences between energy regulation in normal differentiated cells and cancer cells. Warburg analyzed the ratio of oxidative phosphorylation (OxPhos) to glycolysis in different tissues of cancer cells and normal cells. Glycolysis under aerobic conditions was found to be particularly high in aggressive tumors when compared with benign tumors and normal tissues. These observations led Warburg to propose deficiency in OxPhos and elevated glycolysis as the primary cause of cancer.

The discovery of the double helix by Watson and Crick in 1953 and its implications for the understanding of molecular processes in biology diverted interest away from research into the significance of Warburg's metabolic hypothesis. However, the failure of the genomic approach to provide effective therapies for certain types of aggressive cancer, and recent studies [7] creating a rapprochement of genetic and metabolic views have revived interest in the Warburg hypothesis.

The hypothesis, in its simplest form, asserts that cancer is primarily a disease of metabolic dysregulation: a switch, inducible by various agents-genetic, nutritional and environmental, from an OxPhos pathway to a glycolytic mode of energy processing. This focus on metabolism as the primary cause for the progressive transition from normalcy to malignancy suggests a radically new approach to cancer therapy. The focus is to influence metabolic regulation in cancer cells so that the autonomy, proliferative capacity, invasiveness and metastasis which define the aggressive cancer phenotype, is never attained.

A therapeutic strategy which emphasizes containment rather than annihilation is a radical departure from methods designed to eradicate cancer cells completely from tissues. Therapies based on metabolic interventions involve two complementary programs: the down-regulation of glycolysis and the up-regulation of OxPhos.

According to Warburg the aggressiveness of a tumor derives from the elevation of the glycolytic mode of energy processing. This elevation is considered to be the result of competition between cells utilizing the glycolytic mode, and cells adopting OxPhos. Hence, the principle that underlies these complementary programs of disease control is Darwinian: the modulation of the selective advantage of cells using glycolysis and OxPhos, respectively.

It is well known that ATP generation through glycolysis is less efficient than through mitochondrial respiration. Hence a long-standing paradox is how cancer cells with their metabolic disadvantage can survive the competition with normal cells. In terms of biochemical reactions [9], mitochondrial respiration defects lead to activation of the Akt survival pathway through a mechanism mediated by NADH. Respiration-deficient cells harboring mitochondrial defects exhibit dependency on glycolysis, increased NADH, and activation of Akt, leading to survival advantage and also drug resistance in hypoxia [9].

The efforts to implement these therapeutic programs have generated certain significant questions regarding the analytical characterization of Darwinian fitness, the capacity of a cell type to displace related types in competition for resources. The problem can be formulated as follows: (a) What class of physiological, biochemical and biophysical properties of cells confers a selective advantage during evolution of the cancer phenotype? (b) To what extent can these properties be analytically described in terms of bio-energetic and kinetic variables associated with the regulatory circuits that describe the metabolic networks?

These questions are consistent with the view that cancer development proceeds according to an evolutionary process in which a succession of genetic and epigenetic changes, due to the selective advantage conferred, leads to the progressive transformation of normal cells to cancer cells [10-12]. The resolution of the problems addressed in (a) and (b) would evidently provide an analytic framework for cancer therapy based on containment rather than eradication. The analysis would also yield a rationale based on natural selection for Warburg's hypothesis, and consequently, an evolutionary understanding of the origin of cancer.

The analytical framework for a theory of metabolic regulation in cells capable of addressing problems of cellular adaptation and somatic evolution within living organism was proposed in a series of articles [13,14], and called Quantum Metabolism in view of the quantum mechanics methodology the theory invoked. Quantum Metabolism gives a molecular level explanation of certain empirically derived allometric laws relating metabolic rate with cell size.

The allometric rules are of the form [14]:(1)

Here, *P *is the metabolic rate, the rate of ATP production, W the cell size. The dimensionality parameter *d *in the scaling exponent, *β *= *d*/(*d*+1), describes the number of degrees of freedom of the enzymes which catalyze the redox reactions within the energy transducing organelles: mitochondria (in the case of OxPhos), metabolosomes (in the case of glycolysis). The proportionality constant, *α*, depends on the mode of coupling between the electron transport chain and ADP phosphorylation. This mode of coupling is electrical in the case of OxPhos and chemical in the case of glycolysis.

A mathematical theory of evolution by natural selection which, for the first time, considered the effect of resource constraints and finite population size on the outcome of competition between related types, was described in [15]. The cornerstone of this model was the statistical parameter, evolutionary entropy, a measure of the stability of population numbers, and an index of Darwinian Fitness.

In this article we will integrate Quantum Metabolism with certain analytic relations between evolutionary entropy and metabolic rate to show that selective advantage in cellular evolution is contingent on the resource constraints - its abundance and distribution and predicted by the metabolic rate, the rate at which cells transform resources into metabolic work. Quantum Metabolism predicts that the metabolic rate of cells utilizing OxPhos and cells utilizing glycolysis will have the same scaling exponents but will differ in terms of the proportionality constants. We will exploit this prediction, and the characterization of selective advantage in terms of resource constraints and metabolic rate, to provide an evolutionary rationale for the cancer phenotype. The evolutionary argument rests on differences in the metabolic rate of cells utilizing OxPhos and glycolytic pathways, respectively. Cellular metabolic rate can be influenced by perturbing the geometry of the metabolic network or the mode of coupling hence the incidence of OxPhos and glycloysis in normal and cancer cells can be metabolically regulated. We will appeal to these notions of metabolic intervention to propose anti-cancer strategies based on arresting the transformation from a benign tumor, to a malignant tissue characterized by enhanced glycolysis.

This article will give a brief account of Quantum Metabolism and the scaling laws for metabolic rate which the new theory derives. We discuss the evolutionary perspective this theory entails, and then apply the new class of models to propose therapies based on altering the selective advantage of normal and cancer cells during the transition to the cancer phenotype.

### Quantum metabolism

Cellular metabolism is the totality of all chemical reactions in cells carried out by an organism. The characteristics of living organisms, such as their growth, the maintenance of their structure and mass transport, depend on the input of energy from the environment. Metabolism designates the series of chemical reactions that transform substrates such as glucose into cellular building blocks, and energy in the form of ATP. A quantitative understanding of the rules which regulate this energy transformation is critical for a quantitative characterization of the selective advantage associated with different modes of energy processing.

Quantum Metabolism exploits the methodology of the quantum theory of solids, as developed by Einstein and Debye, to derive a class of analytic rules relating metabolic rate, with cell size. The variables which define these rules are bio-energetic parameters, whose values depend on the phospholipid composition of the bio-membranes, and enzymatic reaction rates, which depend on the concentration of substrates in metabolic reactions.

#### The allometric laws of metabolism

Metabolic rate, the rate at which an organism transforms nutrients into thermal energy and biological work required for sustaining life, is highly dependent on the organism's size. This observation draws in large part from the experimental studies of Lavoisier and Laplace who were the first to demonstrate the relationship between combustion and respiration. The systematic empirical study of the relation between metabolic rate, P, and body size, W, which began with Rubner [16], and was extended later by Kleiber [17] for non-domesticated mammals, and by Hemmigsen [18] to uni-cells, led to a set of allometric laws relating metabolic rate with body size.

Quantum Metabolism exploits the formalism of quantum mechanics to study the dynamics of energy flow in the electron transfer chain in cells, and provides a molecular level explanation of the empirical rules documented in Kleiber [17] and Hemmingsen [18].

The derivation of Eq. (1) is based on the assumption that the transformation of nutrients into thermal energy and biological work involves the inter-conversion of two forms of energy [19,20]:

(1) The redox potential difference, that is the actual redox potential between the donor and acceptor couples in the electron transport chain.

(2) The phosphorylation potential for ATP synthesis.

The parameter d in the scaling exponent *β *= *d/(d+1)*, characterizes the number of degrees of freedom of the enzymes which are embedded in the energy transducing organelles. Enzymatic reactions have an intrinsic direction and the enzymes localized in the organelles have a given orientation. The enzymes are subject to oscillations, due to the redox potential. We will assume that these vibrations can be approximated by harmonic oscillators. We will also assume that the enzymatic vibrations are coupled and inherited by the energy transducing organelles in which the enzymes reside.

There exists a diverse body of empirical support for these assumptions. Some of this support can be annotated as follows. Experimental studies of the organization of mitochondrial networks show that these systems can be regarded as coupled oscillators [21]. Synchronisation of metabolic cycles through gene and enzyme regulation within and between cells has been shown to involve co-ordinated transcriptional cycles not only in cultured yeast but also importantly in mammalian cells [22-24]. Tsong and collaborators have demonstrated that dynamical processes govern the function of metabolic enzymes which can capture and transmit energy from oscillating electric fields [25] involving electro-conformational coupling [26] and electric modulation of membrane proteins [27].

Our model rests on the hypothesis that the metabolic energy of the cell is characterized by the coupled oscillations of the energy transducing organelles. Consequently, there are three levels of metabolic organization to be considered: (a) the energy contained in the vibrations at the level of individual enzymes, (b) the coupling of the enzymes within the organelles, and (c) the coupling of the energy transducing organelles within the cell.

Because the energy which drives the process of metabolic regulation depends on coupling at two distinct levels, the enzymatic level and the level of the energy transducing organelles, we can assume that the dimensionality parameter d will depend on physico-chemical properties of the cellular matrix at: (a) the level of the enzymes, (b) the level of the mitochondria and metabolosomes. Hence, we can assume that d, an index of the number of degrees of freedom of the enzymes aligned in the organelles, may not satisfy the condition 1<d<3, as in the Debye model, but may vary in principle between 1 and infinity.

#### Cycle time and metabolic rate

Quantum Metabolism establishes that the mode of energy transfer may either be quantized or classical, contingent on: (a) the cycle time, *τ*, of the metabolic processes transforming substrates into products within the network of chemical reactions, and (b) the relaxation time, τ *, which describes the return time of enzyme concentrations to their steady state condition after a random perturbation.

The mean cycle time, *τ*, is highly dependent on the external resources, their concentration and diversity, whereas the relaxation time, τ *, depends on the density of the metabolic enzymes and the physical properties of cellular medium [14].

The mean cycle time is the mean turnover time of the enzymes in the reaction process. Based on typical data for ion pumps in biological membranes, the range of values expected for *τ *is between 10^-6^s and 10^-3^s [27,28]. Recently, the proton-driven ATP synthase rotor has been reported to have Ohmic conductance on the order of 10 fS [29]. Hence, we can estimate for this value the number of protons involved in each cycle to range from 10 at the high frequency limit to 10, 000 at the low-frequency limit. More recent papers provided a novel description of mitochondria as individual oscillators whose dynamics may obey collective, network properties in terms of high-amplitude, self-sustained and synchronous oscillations of bio-energetic parameters under both physiological and patho-physiological conditions [30]. This was demonstrated through the analysis of their power spectrum that exhibits an exponent vastly different from random behavior. Therefore, a proposed description of the metabolic activity involving mitochondrial proteins as coupled quantum oscillators of the Debye type appears to be supported by recent observations.

According to Quantum Metabolism, the quantized or classical modes of energy transfer are determined by the extreme values assumed by the ratio *τ*/τ*. We have the following two scenarios [14]:

(a) When *τ *<<τ*, the mode of energy transfer is discrete and the dynamic of energy flow is described by quantum laws. In this case d is finite and the scaling exponent, *β*, satisfies 1/2 <*β *< 1.

(b) When *τ *>>τ*, energy transduction is continuous and the dynamic of energy transfer is described by classical laws. Here, the parameter d is infinite and the scaling exponent *β *= 1.

#### Proportionality constant and metabolic rate

The proportionality constant, *α*, is determined by the mechanism - electrical or chemical - which describes the coupling between electron transfer and ATP assembly. The coupling between the electron transport chain and ADP phosphorylation is electrical, in the case of OxPhos, and chemical in the case of glycolytic processes. The metabolic rate in cells utilizing each of these processes will differ, the difference being due to the proportionality constant.

*(I) Oxidative phosphorylation*. OxPhos is a mode of energy processing whereby the cell carries out phosphorylation of ADP during the oxygen-dependent transmission of electrons down the respiratory chain in the relevant membrane. This reaction produces NADH which then fuels OxPhos to maximize ATP production with minimal production of lactate. The coupling between the electron transport chain and ADP phosphorylation is generated by the flow of protons across the bio-membrane. The proportionality constant *α *is a function of the bio-energetic parameters, proton conductance *C*, and the proton-motive potential *Δp*. These parameters will depend on the phospholipid composition of the membrane [31].

(IIa) *Anaerobic glycolysis *(glycolysis which is suppressed by oxygen). Anaerobic glycolysis describes a mode of energy generation when oxygen supply is limited. In this process, cells direct the pyruvate generated by glycolysis away from the mitochondrial OxPhos pathway. This is achieved by generating lactate. This process does not occur within the mitochondria but in the liquid protoplasm of the cell. This generation of lactate allows glycolysis to continue but results in a lowered ATP production rate. The rate-limiting factors for net ATP production are the efficiencies of glucose delivery and lactate removal.

(IIb) *Aerobic glycolysis *(glycolysis, which is not suppressed by oxygen). Aerobic glycolysis refers to the conversion of glucose to pyruvate and then to lactate regardless of whether oxygen is present or not. This process is also localized in the liquid cytoplasm. This property is shared by normal proliferative tissue.

The proportionality constant in both aerobic and anerobic glycolysis is a function of the activity of the glycolytic enzymes.

We wish to mention that the terms aerobic and anaerobic glycolysis are imprecise, although they have been used extensively in the literature (see for example [12]). The term aerobic glycolysis is somehow misleading since both types of glycolysis are in fact anaerobic. Warburg used the term aerobic glycolysis to emphasize the important difference from normal glycolysis that it is not suppressed by oxygen. Any type of glycolysis is always substantially the same pathway. It runs much faster in the absence than in the presence of oxygen because of feedback regulation. For example, when oxygen is available, the highly efficient ADP-phosphorylating system in the energy-transducing bio-membrane ensures a high ATP/AMP ratio in the cell, which down-regulates the glycolytic enzymes. The distinction between aerobic and anaerobic glycolysis is therefore only one of net flux rate. Consequently, some researchers consider the distinction redundant. Glycolysis is not oxygen-dependent; but it is *always *(partly) suppressed by oxygen.

#### An empirical observation

Quantum Metabolism predicts that the range of values assumed by the scaling exponent, *β*, *β *< 1 (an allometric relation), *β *= 1 (an isometry), is highly dependent on the cycle time, *τ*, a quantity which varies with the resource flux - an environmentally regulated property. This observation indicates that changes in environmental factors can induce significant changes in the scaling exponent with concomitant changes in metabolic rate. Empirical support for this prediction is given in Nakaya et. al [32]. The experiments in [32], reported a shift from a scaling law with *β *= 3/4. to a scaling law described by *β *= 1, contingent on changes in the environmental conditions. The existence of such environmental switches is particularly pertinent in applications of quantum metabolism to somatic evolution. Such changes in the scaling exponent indicate a mechanism for regulating the metabolic profile of cells by imposing various environmental constraints.

### Oxidative phosphorylation and glycolysis: a bio-energetic comparison

Quantum Metabolism predicts that the scaling laws for cells utilizing OxPhos and glycolysis will be described by similar scaling exponents but different proportionality constants. The proportionality constant is determined by the mode of coupling between the electron transport chain and ADP phosphorylation. In OxPhos coupling is achieved by a single common intermediate between the oxidation of a variety of substrates and ATP formation. This intermediate is the trans-membrane proton gradient. For glycolysis, there is a single set of enzymes for every coupled reaction.

The differences in the mode of coupling entail significant differences in the metabolic efficiency of cells utilizing OxPhos and cells using glycolysis. With glucose as substrate, OxPhos generates about 17 times more ATP than glycolysis. Consequently, the metabolic rate of OxPhos (the respiration rate) will also be greater than the metabolic rate of glycolysis (the fermentation rate). The evolutionary history of the different modes of energy production will provide some perspective on the selective advantage which respiration and fermentation conferred as the environmental conditions and resource constraints changed during the history of life on Earth.

The fermentative way of energy generation is now accepted as the primordial mode of energy processing [33]. The invention of photosynthesis changed the atmosphere, since bacteria and plants used light energy to split water into hydrogen and oxygen. After accumulation of oxygen in the atmosphere, OxPhos as a new way of energy generation was established by bacteria. Such free living bacteria have been integrated into eukaryotic cells and represent the symbiosis of two different cell types. By acquiring bacteria capable of OxPhos a new organism emerged having the choice between energy release based on substrate phosphorylation or OxPhos. During evolution of higher vertebrates duplication and modification of genes [33] led to a fermentative energy generation which is not suppressed by oxygen [8]. Since this type of glycolysis is performed even in the presence of oxygen, Warburg called it aerobic glycolysis. The term aerobic glycolysis is somehow misleading since both types of glycolysis are anaerobic. Warburg's term aerobic glycolysis tries to emphasize the fact that the important difference to normal glycolysis is that it is not suppressed by oxygen.

These observations suggest that in the course of evolution, anaerobic glycolysis arose first. The primitive nature of glycolysis, in contrast to OxPhos, is indicated by the fact that the glycolytic enzymes exist free in solution in the soluble portion of the cytoplasm. This is in sharp contrast to the enzyme systems responsible for respiration and photosynthesis. These enzymes are grouped and arranged in an intracellular structure organized in the mitochondria and chloroplasts [20].

### Modes of energy processing: their incidence

Most cancer cells have an increased glycolysis. However, the relative contribution of glycolysis to ATP supply varies considerably with the tissue. The dependence of glycolytic contribution on tissue type is shown in Table 1[19]. Aerobic glycolysis is also observed in certain normal cells. A selected group is given in Table 2[19].

**Table 1:** Predominant energy metabolism in different types of tumor cells

Tissue of Tumor	Cell Type	Predominant Energy Metabolism
Brain	Glioma	Gly
Bone	Sarcoma	OxPhos
Colon	Colon adenocarcinomas	Gly
Lung	Lung carcinoma	Ox Phos
Skin	Melanoma	Ox Phos

**Table 2:** Glycolytic ATP contribution in selected normal cell types.

Cell Type	Percentage of Glycolytic ATP contribution
Mouse macrophages	18
Pig platelets	57
Rat coronary endothelial cells	53
Human platelets	24

In humans, aerobic glycolysis is extremely important in cells like neurons, retinal cells, stem cells and germ cells. Neurons, for example, depend absolutely on glucose as an energy source and cannot function anaerobically. The utility of anaerobic glycolysis, to a muscle cell for example, when it needs to utilize large amounts of energy in a short period of time, stems from the fact that the rate of ATP production from glycolysis is approximately 100 times faster than from oxidative phosphorylation. During exertion muscle cells do not need to activate anabolic reaction pathways. The requirement is to generate the maximum amount of ATP, for muscle contraction, in the shortest time frame. This is the reason why muscle cells derive almost all of the ATP consumed during exertion from anaerobic glycolysis. As these types of cells do not display the cancer phenotype, it is evident that elevated glycolysis is not a sufficient condition for tumorigenesis.

Elevated glycolysis is also not always observed at all stages of tumorigenesis. The studies reported in de Groof et. al. [34] based on transformed fibroblasts in mice, indicate the multi-step nature of the progression from normalcy to the malignant state. These studies document a high rate of OxPhos in newly transformed cells with a subsequent high glycolytic rate in the malignant state. The experimental system shows that the proliferation of malignant cells hinges on the evolution of different metabolic profiles as an adaptive response to the changes in the resource conditions during the transition from normal to malignant cells.

We will now show how these evolutionary changes in metabolic profile can be understood in terms of a measure of selective advantage - a prescription derived from Quantum Metabolism and evolutionary dynamics.

### Darwinian fitness in somatic evolution

The concept Darwinian fitness describes the capacity of a variant type to increase in frequency in competition with an incumbent population for the available resources. This notion is of fundamental importance in all analytical studies of the Darwinian process at molecular, cellular and organismic levels.

Studies of selective advantage in evolutionary processes were pioneered by Fisher [35], who proposed the population growth rate, denoted r, as the measure of Darwinian fitness. According to Fisher, selective advantage in evolutionary dynamics is given by(2)

Here, Δ*r *= r* - *r*, where r* is growth rate of the variant and incumbent type, respectively. These models have provided qualitative insight into several studies of the evolutionary process, and since 1930 have been the dominant approach in evolutionary genetics - in spite of several inconsistencies in their predictive and explanatory power.

#### Measure of selective advantage: evolutionary entropy

The analytical and conceptual basis for the limitations of the classical models proposed by Fisher, was only recently recognized. The studies reported in [15] showed that the population growth rate as a measure of fitness is only valid when population size and resources are infinite. Consequently, the Malthusian parameter will only be a meaningful selective index when population sizes are large.

The studies initiated by Demetrius [15] and later developed as a general model of the evolutionary process showed that the outcome of competition between an incumbent and a variant type is conditional on the magnitude and the variation in resource constraints, and is predicted by the robustness or the demographic stability of the population [36]. Robustness describes the rate at which the population returns to its steady state condition after a random perturbation in the individual birth and death rates. This property can be analytically described in terms of the quantity evolutionary entropy, an information theoretic measure which describes the uncertainty in the state - age, size, metabolic energy - of the immediate ancestor of a randomly chosen newborn. Evolutionary entropy in cellular populations describes the variability in the rate at which individual cells pass through the various stages of the cell cycle. A synchronous population has small entropy, an asynchronous population has large entropy.

Selective advantage in the context of this model is given by(3)

Here, Δ*S *= S* - *S*, where S* and *S *represent the entropy of the variant and the incumbent type, respectively. The quantity *Φ*, the reproductive potential, and *γ*, the demographic index, are statistical parameters which depend on the survivorship and replication rate of the cells in the population. These statistical parameterscharacterize certain measures of resource constraints, assuming that the changes in the resource conditions are in dynamical equilibrium with changes in the number of cells. The parameter *Φ *describes mean resource abundance and *γ *the variance in the resource abundance.

(i) *Φ *< 0 corresponds to limited resource

(ii) *Φ *> 0 corresponds to unlimited resource

and

(iii) *γ *< 0 corresponds to a variable resource distribution

(iv) *γ *> 0 characterizes a constant resource distribution

The measure of selective advantage given by Eq. (3) is a far-reaching generalization of the measure given by Eq. (2). Indeed, Eq. (3) reduces to Eq. (2) when *γ *= 0 or when the population size *N *tends to infinity. The condition *γ *= 0 corresponds to a complete correlation between the resource variability and the demographic variability.

In view of the characterization of the statistical parameters *Φ *and *γ *as resource constraints, the measure of selective advantage given by Eq. (3) can be qualitatively described in terms of the following two tenets.

(Ia) *When resources are constant and limited, variants with increased entropy will have a selective advantage and increase in frequency in competition with the resident type*.

An **increased **entropy describes an increased capacity to maintain a constant population size when subject to random perturbations in the individual birth and death rates. This stability condition confers a selective advantage when resources are limited.

(Ib) *When resources fluctuate in abundance, variants with decreased entropy will have a selective advantage and increase in frequency in competition with the resident type*.

A **decreased **entropy represents an increased sensitivity of population numbers to random perturbations in the individual birth and death rates. This increased sensitivity - which entails a decreased resilience or stability - now confers a selective advantage when resources fluctuate in abundance, as it enables the population to track the changes in the resource abundance.

The importance of this measure of selective advantage for the study of cellular evolution and the evolution of the cancer phenotype rests on the fact that evolutionary entropy, a population parameter, is analytically related to physiological and morphometric properties of individual cells [37]. Consequently, the selective advantage can also be described in terms of changes in individual properties such as metabolic rate and cell size.

### Metabolic rate as fitness

The central result of quantum metabolism is the allometric relation between metabolic rate and cell size given by Eq. (1). An immediate consequence of this allometric law is a scaling rule involving cycle time, *τ*, and cell size W. This is given by(4)

A fundamental result in studies of the macroscopic parameters that characterize the population dynamic is the relation between evolutionary entropy *S *and the cycle time, *τ*, see Demetrius, Legendre and Harremöes [37], namely:(5)

where *a*_1 _is a taxon specific constant. Eqs. (1), (4) and (5) can be combined to derive a relation between metabolic rate *P *and evolutionary entropy, namely:(6)

where the taxon-specific constants *a*_2 _and *b*_2 _are positive.

We can therefore infer that evolutionary changes in entropy and metabolic rate will be positively correlated. This entails that the selective advantage given by Eq. (3) can now be expressed in the form:(7)

This result asserts that the outcome of competition between a resident and a variant cell type will be dependent on the resource abundance, as described by *Φ*, the resource variability, as expressed by *γ*, the population size, *N*, and the metabolic rate *P*.

The significance of metabolic rate in the description of selective advantage, as given by Eq. (7), revolves around the observation that cellular metabolic rate is constrained by two main factors: (a) the rate at which the cells acquire resources, and (b) the rate at which resources acquired are transformed into cellular work.

In this context, when population size, *N*, is large, the rules describing the outcome of competition between resident and variant types which Eq. (7) prescribes, can be qualitatively annotated in the following terms.

(IIa) *When resources are constant and limited, variants with increased metabolic rate have a selective advantage and increase in frequency in competition with the resident type*.

An increased metabolic rate entails an increased efficiency in the acquisition of resources. This condition will confer a selective advantage when resources are limited. Hence, when limited resource conditions prevail, types with higher metabolic rates will be favoured.

(IIb) *When resources vary in abundance, and the mean abundance is large, resource density is no longer a limiting constraint and types with decreased metabolic rate will have a selective advantage and increase in frequency*.

When the mean abundance is large, selective outcome will no longer be contingent on the efficiency in resource acquisition. The dynamics of selection will now be determined by the capacity of cells to transform resources into cellular work. In view of the allometric relations between metabolic rate and cell size, cells with decreased metabolic rates will typically have smaller sizes; consequently their capacity to transform resources into cellular activity will be enhanced. Therefore, under abundant resource conditions cells with decreased metabolic rate will prevail.

These general rules provide a basis for predicting changes in metabolic profile-metabolic rate and metabolic efficiency - as one cell type replaces a related type by natural selection.

Metabolic rate is the rate of ATP production per unit time. Metabolic efficiency is the amount of ATP produced per unit of substrate material. The metabolic rate is the minimum rate at which the cell uses energy to stay alive. All living systems exist in a steady state relatively far from thermodynamic equilibrium and this state is maintained by sustaining non-equilibrium concentration gradients across membranes. This activity requires a constant influx of metabolic energy, and the consequent generation of entropy. The ratio of the energy stored in the various bio-molecules to the free energy released in redox reactions is the efficiency of the metabolic process, which can be as high as about 95% in the case of the Krebs cycle. This high efficiency is characteristic of cyclic processes.

Our analysis rests on certain critical differences in the metabolic rate of cells utilizing fermentation and respiration. First, we observe that there are significant differences in the metabolic efficiency between glycolysis and OxPhos. The metabolism of glucose to lactate generates only 2 ATP (net) per molecule of glucose, whereas OxPhos generates up to 38 ATP molecules (net) [38]. Now, metabolic efficiency and metabolic rate in cells are determined by the same processes and the same reactions. Consequently, metabolic rate under glycolysis will be less than that under OxPhos. These observations, together with the expression for selective advantage given by Eq. (7), imply that the metabolic profile - fermentation or respiration which defines a cell type will be determined by the resource constraints the populations experience during their evolutionary history.

We can, therefore, infer from the principles described in (IIa) and (IIb) the following rules relating resource constraints and the selective advantage of the two classes of metabolic pathways.

(IIIa) *When resources are constant and limited, cells utilizing oxidative phosphorylation will have a selective advantage*.

(IIIb) *When resources vary in abundance and the mean abundance is large, cells utilizing the glycolytic pathway will have a selective advantage*.

These principles, which derive from the higher metabolic efficiency and concomitantly the higher metabolic rate of OxPhos, provide an evolutionary explanation of the mode of energy processing observed in microbes and higher organisms. The principles given by (IIIa) and (IIIb) entail that microbes and higher organisms will be characterized by the OxPhos mode of energy processing when evolving under limited resource conditions, and a glycolytic mode when evolving under exponential growth conditions.

Empirical observations on the metabolic phenotypes of microbes and cells from multi-cellular organisms are consistent with these predictions. Unicellular organisms starved of nutrients respire aerobically, as is the case of cells in multi-cellular organisms that are maintained in a non-proliferative state. Unicellular organisms undergoing exponential growth metabolize glucose by fermentation into a small organic molecule such as ethanol. Proliferating cells in multi-cellular organisms exhibit a similar metabolic profile: these cells utilize glycolysis and excrete large amounts of lactate.

The selection principle based on metabolic rate can also be cited to explain the use of aerobic glycolysis in certain classes of cells, namely, neurons, retinal cells, stem cells and germ cells. Aerobic glycolysis confers a selective advantage in cells evolving under conditions where the resources are abundant. Neurons, retinal cells and stem cells require for their metabolic functioning, a large resource energy. The presence of glycolysis as the mode of energy processing in these cells is the selective outcome of the environmental conditions under which these types of cells evolve. These cells do not develop the cancer phenotype since the energy which the cells absorb is constantly being used to maintain the homeostatic integrity of the organism.

### The Warburg Effect: Competition between cancer cells and normal cells

Quantum Metabolism predicts that differences in the metabolic rate of cells using OxPhos and cells using glycolysis derive primarily from the differences in the mode of coupling between the energy generated by the flow of electrons in the electron transport chain and ADP phosphorylation.

In OxPhos, the coupling is electrical and the metabolic rate is determined by the flow of protons across the mitochondrial inner membrane. In glycolysis, the coupling is chemical and the metabolic rate is regulated by the activity of the glycolytic enzymes.

Carcinogenesis is a multi-step process that implicates the activation of oncogenes, the inactivation of tumor suppressor genes and the ultimate emergence and proliferation of the cancer phenotype. The transition to the glycolytic phenotype of malignant cells which define the Warburg effect is a consequence of an evolutionary process. Our synthesis of Quantum Metabolism [14] and evolutionary dynamics [15] establishes that the index of selective advantage which describes this process is a function of the resource constraints and the metabolic rate of the competing cell types.

The measure of selective advantage given by Eq. (7) entails that the outcome of competition between normal cells, metabolizing the OxPhos pathway and cells using glycolysis, is a stochastic event contingent on the resource density and distribution. The relation between resource constraints and selective outcome is a consequence of Eq. (7) and can be summarized in Table 3.

**Table 3:** Outcomes of competition between resident population using OxPhos and mutant cells using glycolysis.

Resource Constraints	Selective Outcome
(I) Limited resources	Ox Phos [(almost always) = (a.s.)]
Constant distribution	
(II) Abundant resources	
Variable Distribution	Glycolysis (a.s.)
(III) Limited Resources	
Variable distribution:	
Large population size	Ox Phos (a.s.)
Small population size	Ox Phos (with probability that increases with population size)
(IV) Abundant resources	
Constant distribution	
Large population size	Glycolysis (a.s.)
Small population size	Glycolysis (with probability that increases with population size)

Table 3 underscores the stochastic nature of the outcome of competition between the two types of cells and consequently the stochastic nature of the evolution of the glycolytic phenotype. The selective outcome depends not only on the resource constraints but also on the population size of the cells from which the tumor originated. Items (III) and (IV) in Table 3 indicate that when the population size is small, the selective outcome is highly stochastic - a glycolytic phenotype may outcompete the OxPhos pathway, even though the resources are limited; and an OxPhos phenotype may prevail in competition even though the resources are fluctuating. The stochasticity which small population size generates - a property emphasized in the general theoretical study in [36] - indicates that when resources are limited, tumor cells may even invade a population of normal differentiated cells.

The stochasticity which Table 3 delineates partly explains the non-universality of the Warburg effect and the caution which must be exercised in using the effect as a basis for detecting cancer. Most but not all cancer cells display the glycolytic phenotype.

### Therapeutic Strategies

The Warburg hypothesis asserts that cancer is primarily a disease of metabolic dysregulation. The activation of oncogenes and the inactivation of tumor suppressor genes have engineered alterations in metabolism and a shift from an oxidative mode of energy processing to a glycolytic mode [38].

This phenomenon suggests that therapeutic strategies based on arresting the transition from a normal differentiated cell defined by oxidative mode of energy processing, to a malignant cell defined by up-regulation of glycolysis, may be effective in complementing traditional methods based on radiation and chemotherapy.

The evolutionary rationale for the shift to the up-regulation of the glycolytic pathway we have delineated, has provided an understanding of the forces and events which drive cells towards malignancies. Our analysis has implicated several factors:

(1) The density and variability of the resources the cells utilize.

(2) The population size of the tumor cells.

(3) The relative metabolic rate of the normal and tumor cells.

Our evolutionary study has discovered two critical features of the competitive dynamics of tumor cells and their normal ancestors.

(1) When resources are constant and limited, the OxPhos phenotype, in view of its higher metabolic rate, will have a selective advantage. Consequently, cells displaying the glycolytic phenotype will have a relatively low probability of becoming established and proliferating. In essence, limited resource condition will inhibit the proliferation of the cancer phenotype.

(2) When resources vary in abundance, the glycolytic phenotype, in view of its lower metabolic rate, will have a selective advantage. Accordingly, cells displaying the glycolytic phenotype will have an enhanced probability of becoming established. Hence resources which show large variation in abundance will facilitate the development of the cancer phenotype, its invasiveness and metastases.

These observations suggest at least four distinct classes of therapeutic strategies to contain the shift of tumors towards the malignant state. These strategies are based on various types of metabolic interventions, some of which have been extensively investigated in the past oblivious, however, to the theoretical rationale which quantum metabolism confers.

#### Modifying the metabolic rate of cancer cells

Cancer cells exploit the glycolytic pathway as the mode of energy processing. The metabolic rate of cells using this mode of energy processing is determined primarily by the glycolytic enzymes [39]: hexokinase HXK, phosphofructokinase, (PFK), lactate dehydrogenase, (LDH), transketolase (TKTL1). These enzymes are generally up regulated in various cancer cells. This class of enzymes represent potential therapeutic targets as the modification of their activity will have significant effects on the metabolic rate of cancer cells and hence their selective advantage.

It is worth noting in this connection that the proton-motive force across a membrane, Δp, is given by [36](8)

where ΔΨ is the membrane potential, F denotes Faraday's constant and ΔpH is the change in pH across the membrane. The values of Δp, ΔΨ and ΔpH depend on the geometry and chemical composition of the energy transducing membrane. Consequently, the metabolic rates can be controlled by not only the membrane composition and its geometry but also the variations in temperature and pH. One of the well-known characteristics of cancer cells is their difference in pH (acidic) compared to normal cells. Interestingly, cancer cells appear to exhibit greater heat sensitivity than normal cells which may also lead to therapeutic applications. Recent biochemical and clinical studies have revealed a profound and selective toxic effect of elevated temperatures on tumor cells [40].

#### Modifying the metabolic rate of normal cells

The metabolic rate of normal cells is primarily determined by the proton conductance and the proton potential of the metabolic systems. These bio-energetic parameters are regulated by the phospholipid composition of the mitochondrial membrane. This composition can be modified by exercise and diet [41]. Such modification can increase the metabolic rate of normal cells, thus enhancing its selective advantage, under limited resource conditions, with cells utilizing the glycolytic pathway.

It is also tantalizing to speculate about the high therapeutic value of metabolism-oriented drugs, such as the recently headlined molecule dichloroacetic acid (DCA) which attempts to restore OxPhos and hence mitochondrial function, ultimately restoring apoptosis which has been demonstrated to kill cancer cells *in vitro*, and shrink tumors in rats [42]. DCA is currently in clinical trials and it is hoped that it may become the first effective drug in dealing with the glycolytic pathway activated in cancers. A recent report [34] has shown that inhibition of glycolysis severely depletes ATP in cancer cells, especially in clones of cancer cells with mitochondrial respiration defects, and leads to rapid dephosphorylation of the glycolysis-apoptosis integrating molecule BAD, and cell death. Importantly, inhibition of glycolysis effectively kills colon cancer cells and lymphoma cells in a hypoxic environment in which the cancer cells exhibit high glycolytic activity and decreased sensitivity to common anticancer agents.

#### Changing the cycle time using uncoupling agents and inhibitors

Cycle time can be experimentally manipulated, for instance, using uncoupling agents and inhibitors of electron transport. There are six distinct types of poisons which may affect mitochondrial function [39]: (i) respiratory chain inhibitors (e.g. cyanide, antimycin, rotenone, TTFA, malonate) which block respiration in the presence of either ADP or uncoupling agents; (ii) phosphorylation inhibitors (e.g. oligomycin) which abolish the burst of oxygen consumption; (iii) uncoupling agents (e.g. DNP, CCCP, FCCP, oligomycin) which disrupt the linkage between the respiratory chain and the phosphorylation system; (iv) transport inhibitors (e.g. atractyloside, bongkrekic acid, NEM) which either prevent the export of ATP, or the import of raw materials across the mitochondrial inner membrane; (v) ionophores (e.g. valinomycin, nigericin) which make the inner membrane permeable to compounds which are ordinarily unable to cross it; and (vi) Krebs cycle inhibitors (e.g. arsenite, aminooxyacetate) which block one or more of the TCA cycle enzymes, or an ancillary reaction.

According to Quantum Metabolism, changing the cycle time can induce a change of the scaling exponent *β *[14] which, in turn, will affect the metabolic rate of the cell type. This can further alter the selective advantage of tumor cells and thereby impede the transition to a malignant state. However, the toxicity of these compounds makes clinical applications to cancer therapy very challenging, except perhaps for the arsenic compounds. Hence novel uncoupling agents are still much in demand.

## Discussion and Conclusions

These differences in the factors that determine metabolic rate in the two modes of energy processing have been exploited to explain the Warburg effect and the selective advantage of the glycolytic phenotype. The understanding of the metabolic basis of carcinogenesis which this evolutionary study generates suggests new methods for regulating cellular metabolic rate and thereby impeding the transition from a benign tumor to a malignant state. In this connection, it is interesting to note that Western diet, which is very calorie-rich, confers evolutionary advantage to cancer cells, hence the increased incidence of cancer in North America and Europe compared to developing countries. Furthermore, increased longevity contributes to this effect as well, since it is well-known that metabolic rate decreases with age of individuals, and in the West the caloric intake does not fall with age at the same rate, thus providing an additional factor for the growth of tumors.

Quantum metabolism, a new bio-energetic theory of metabolic regulation in cells, shows that the proportionality constants in the scaling laws for metabolic rate of cells utilizing OxPhos and glycolysis pathways are contingent on the different modes of coupling between the electron transfer chain and ADP phosphorylation.

(1) In OxPhos, the coupling between the redox reactions (in the mitochondria) and ADP phosphorylation is electrical. Hence the metabolic rate is determined by bio-energetic parameters such as the proton conductance and proton potential of the metabolic network.

(2) In glycolysis, ADP phosphorylation is linked chemically to individual enzyme-catalyzed reactions, so the rate of ATP production is a function of the flux through the pathway as a whole.

Detailed analysis of internal regulation of ATP turnover through both glycolysis and oxidative phosphorylation in terms of specific biochemical mechanisms has been presented in [43,44] in the context of normal cells. Metabolic interventions which focus on modulating the efficiency of these modes of coupling should provide a general framework for regulating the incidence of these two modes of energy processing - and thereby yield a new paradigm for cancer therapy [45]. A recent article [46] has advocated a combination therapy utilizing vitamins as a strategy aimed at energy modulation in cancer leading to the prevention of life-limiting cachexia.

## Competing interests

The authors declare that they have no competing interests. One of the authors, Johannes Coy, declares a potential conflict of interest due to the possible utilization of TKTL1 for diagnostic and/or therapeutic purposes.

## Authors' contributions

LAD developed the evolutionary rationale for the Warburg effect.

LAD and JAT applied the concepts of Quantum Metabolism to the physiological properties of cancer and normal cells. JFC provided specific information regarding therapeutic approaches to the regulation of metabolic pathways. All authors read and approved the final manuscript.
